# Enhancing delirium awareness in South America: current insights and future perspectives for research and practice

**DOI:** 10.31744/einstein_journal/2024CE1332

**Published:** 2024-11-21

**Authors:** Ricardo Kenji Nawa, Thiago Junqueira Avelino-Silva, Roberta Esteves Vieira de Castro, María Adela Goldberg, Luis Daniel Umezawa Makikado, Fernando Tirapegui Sanhueza, Gabriel Heras-La-Calle, Heidi L. Lindroth, Keibun Liu, Rebecca von Haken, Peter Nydahl

**Affiliations:** 1 Hospital Israelita Albert Einstein Department of Critical Care Medicine São Paulo SP Brazil Department of Critical Care Medicine, Hospital Israelita Albert Einstein, São Paulo, SP, Brazil.; 2 Hospital Israelita Albert Einstein Faculdade Israelita de Ciências da Saúde Albert Einstein São Paulo SP Brazil Faculdade Israelita de Ciências da Saúde Albert Einstein, Hospital Israelita Albert Einstein, São Paulo, SP, Brazil.; 3 University of California School of Medicine San Francisco CA USA Division of Geriatrics, School of Medicine, University of California, San Francisco, CA, USA.; 4 Universidade de São Paulo Faculdade de Medicina Hospital das Clínicas São Paulo SP Brazil Laboratório de Investigação Médica em Envelhecimento (LIM-66), Serviço de Geriatria, Hospital das Clínicas, Faculdade de Medicina, Universidade de São Paulo, São Paulo, SP, Brazil.; 5 Universidade do Estado do Rio de Janeiro Hospital Universitário Pedro Ernesto Department of Pediatrics, Pediatric Intensive Care Unit Rio de Janeiro RJ Brazil Department of Pediatrics, Pediatric Intensive Care Unit, Hospital Universitário Pedro Ernesto, Universidade do Estado do Rio de Janeiro, Rio de Janeiro, RJ, Brazil.; 6 Instituto D’Or de Pesquisa e Educação Department of Pediatrics Rio de Janeiro RJ Brazil Department of Pediatrics, Instituto D’Or de Pesquisa e Educação (IDOR), Rio de Janeiro, RJ, Brazil.; 7 Ciudad Autónoma de Buenos Aires Sanatorio de la Trinidad Mitre Buenos Aires Argentina Sanatorio de la Trinidad Mitre, Ciudad Autónoma de Buenos Aires, Buenos Aires, Argentina.; 8 Ciudad Autónoma de Buenos Aires Sociedad Argentina de Terapia Intensiva Buenos Aires Argentina Sociedad Argentina de Terapia Intensiva, Ciudad Autónoma de Buenos Aires, Buenos Aires, Argentina.; 9 Universidad Peruana Cayetano Heredia Intensive Care Unit at Hospital Nacional Cayetano Heredia Lima Peru Universidad Peruana Cayetano Heredia, Intensive Care Unit at Hospital Nacional Cayetano Heredia, Lima, Peru.; 10 Unidad de Paciente Crítico Adulto Complejo Asistencial Dr. Victor Rios Ruiz Los Angeles Chile Unidad de Paciente Crítico Adulto, Complejo Asistencial Dr. Victor Rios Ruiz, Los Angeles, Chile.; 11 Intensive Care Unit of Hospital Universitario de Jaén Madrid Spain Intensive Care Unit of Hospital Universitario de Jaén, Director of the International Research Project for the Humanization of Intensive Care Units (Proyecto HU-CI), Madrid, Spain.; 12 Department of Nursing, Mayo Clinic Division of Nursing Research Rochester MN USA Division of Nursing Research, Department of Nursing, Mayo Clinic, Rochester, MN, USA.; 13 Indiana University School of Medicine Center for Health Innovation and Implementation Science Indianapolis IN USA Center for Aging Research, Regenstrief Institute, Center for Health Innovation and Implementation Science, School of Medicine, Indiana University, Indianapolis, IN, USA.; 14 Non-Profit Organization ICU Collaboration Network Tokyo Japan Non-Profit Organization ICU Collaboration Network (ICON), Tokyo, Japan.; 15 University Hospital Mannheim Department of Surgery Mannheim Germany Department of Surgery, University Hospital Mannheim, Mannheim, Germany.; 16 University Hospital Schleswig-Holstein Nursing Research Kiel Germany Nursing Research, University Hospital Schleswig-Holstein, Kiel, Germany.; 17 Paracelsus Medical University Institute of Nursing Science and Development Salzburg Austria Institute of Nursing Science and Development, Paracelsus Medical University, Salzburg, Austria.

Dear Editor,

With this letter, we aim to supplement the data published by Colleti Junior et al.^(1)^ Evaluating the global prevalence of validated delirium assessment tools and implementing delirium management protocols is essential.^(2)^ Delirium often complicates the care of elderly patients in hospitals, rehabilitation centers, and long-term care facilities, necessitating effective strategies to mitigate its effect on patient outcomes and recovery.^(3)^

The World Delirium Awareness Day (WDAD) study assessed delirium prevalence on March 15, 2023 in 44 countries, 1664 wards, and 36 048 patients. South America contributed to 6.8% of the participation, 1.3% of the wards/units, and data from 0.4% of delirium-assessable patients (Tables 1S to 6S, Supplementary Material).^(2,4)^ Notably, South America demonstrated a high utilization rate of validated delirium assessments at 95.4% (21/22), surpassing the global rate of 61% (15 458/25 268). This trend can be largely attributed to the widespread adoption of the Confusion Assessment Method for the Intensive Care Unit (CAM-ICU) as the most frequently employed assessment tool. The prevalence of delirium in South America was 25.5% (38/149 patients), compared with the global rate of 18% (2788/15 458 patients). Notably, delirium management protocols were implemented in 59.1% (13/22) of wards/units. The top three nonpharmacological interventions for managing delirium cases were mobilization and pain management in 95.5%, and use of bed rails in 90.9%. The most common pharmacological interventions were dexmedetomidine (81.8%), quetiapine (68.2%), and haloperidol (63.6%). Barriers to effective delirium management were reported by 95.5% of respondents and included lack of time to educate staff (59.1%), communication gaps between different professionals (54.5%), and insufficient knowledge about delirium (50%) (Tables 1S to 6S, Supplementary Material).

**Table 1S:** Assessments of delirium cases

Characteristics, n (%)	South America (n=22)	Argentina (n=20)	Chile (n=1)	Peru (n=1)
Validated assessments	21 (95.5)	–	–	–
CAM-ICU	15 (68.2)	13 (65)	1 (100)	1 (100)
Psychiatric consultation	2 (9.1)	2 (10)	–	–
CAP-D	2 (9.1)	2 (10)	–	–
pCAM-ICU	2 (9.1)	2 (10)	–	–
Other assessments	1 (4.5)	1 (5)	–	–
Other	1 (4.5)	1 (5)	–	–

CAM-ICU: confusion assessment method for the intensive care unit; CAP-D: Cornell assessment of pediatric delirium; pCAM-ICU: pediatric confusion assessment method for the intensive care unit.

**Table 2S:** Hospital data

Characteristics		South America (n=22)	Argentina (n=20)	Chile (n=1)	Peru (n=1)
Hospital type, n (%)					
	University hospital	3 (13.6)	2 (10)	–	1 (100)
	University-related/affiliated hospital	4 (18.2)	4 (20)	–	–
	Community hospital	9 (40.9)	8 (40)	1 (100)	–
	Private hospital	6 (27.3)	8 (40)	–	–
Numbers of beds*, n (%)					
	<250	14 (63.6)	13 (65)	–	1 (100)
	<500	7 (31.8)	6 (30)	1 (100)	–
	≥1500	1 (4.5)	1 (5)	–	–
Ward Type, n (%)					
	Emergency department	1 (4.5)	1 (5)	–	–
	High acuity, IMC, ICU	19 (86.4)	17 (85)	1 (100)	1 (100)
	General ward	2 (9.1)	2 (10)	–	–
Discipline, n (%)					
	Medical/nonsurgical	3 (13.6)	3 (15)	–	–
	Respiratory/weaning	6 (27.3)	5 (25)	–	1 (100)
	Mixed/general	10 (45.5)	9 (45)	1 (100)	–
	Rehabilitation	2 (9.1)	2 (10)	–	–
	Other	1 (4.5)	1 (5)	–	–
Age groups, n (%)					
	0-17 years	4 (18.2)	4 (20)	–	–
	18-75 years	13 (59.1)	11 (55)	1 (100)	1 (100)
	>75 years	1 (4.5)	1 (5)	–	–
	Mixed	4 (18.2)	4 (20)	–	–
Number of beds per ward/unit, mean (SD)		15.3 (7.6)	15.3 (7.7)	22	8

* Not reported: <750, < 1000, < 1500 beds.

IMC: intermediate care unit; ICU: intensive care unit; SD: standard deviation.

**Table 3S:** Delirium assessment and management

Characteristics, n (%)	South America (n=22)	Argentina (n=20)	Chile (n=1)	Peru (n=1)
Protocols*, total	112	100	7	5
SBT management#	14 (63.6)	12 (60)	1 (100)	1 (100)
Delirium management (assessment, prevention, and management)	13 (59.1)	11 (55)	1 (100)	1 (100)
Sedation	12 (54.5)	10 (50)	1 (100)	1 (100)
Pain management (assessment, prevention, and management)	10 (45.5)	10 (50)	–	–
SAT management	10 (45.5)	10 (50)	–	–
Nutrition management	9 (40.9)	7 (35)	1 (100)	1 (100)
Mobilization	8 (36.4)	7 (35)	1 (100)	
Family engagement and empowerment	5 (22.7)	4 (20)	1 (100)	–
Physical restraints	4 (18.2)	4 (20)	–	–
Sleep	3 (13.6)	3 (15)	–	–
ICU diaries#	2 (9)	2 (10)	–	–
Other	22 (100)	20 (90)	1 (100)	1 (100)
Delirium awareness interventions, total	38	34	3	1
Delirium mentioned in handovers	15 (68.2)	14 (35)	1 (100)	–
At least one educational training about delirium in the last year	12 (54.5)	10 (50)	1 (100)	1 (100)
Delirium rate and feedback	3 (13.6)	2 (10)	1 (100)	–
Delirium flyer for the staff	3 (13.6)	3 (15)	–	–
Informational posters about delirium	2 (9.1)	2 (10)	–	–
Pocket cards for delirium assessment/management	2 (9.1)	2 (10)	–	–
Delirium experts, known by the team and dedicated for delirium care	1 (4.5)	1 (5)	–	–

* Not reported: protocols for dementia;

# Usually used in intensive care units only. ICU: intensive care unit; SAT: spontaneous awakening trial; SBT: spontaneous breathing trial.

**Table 4S:** Nonpharmacological management

Characteristics, n (%)	South America (n=22)	Argentina (n=20)	Chile (n=1)	Peru (n=1)
Pain management	21 (95.5)	19 (95)	1 (100)	1 (100)
Mobilization	21 (95.5)	19 (95)	1 (100)	1 (100)
Bed boarders	20 (90.9)	19 (95)	1 (100)	–
Family information	18 (81.8)	16 (80)	1 (100)	1 (100)
Physical restraints	17 (77.3)	15 (75)	1 (100)	1 (100)
Cognitive stimulation	15 (68.2)	13 (65)	1 (100)	1 (100)
Open or liberal visiting times	14 (63.6)	14 (70)	–	–
Verbal reorientation	12 (63.6)	11 (55)	1 (100)	–
Provision of glasses, hearing aids, mobility aids	11 (50)	10 (50)	1 (100)	–
Multiprofessional team rounds	11 (50)	9 (45)	1 (100)	1 (100)
Provision of day/night rhythm	8 (36.4)	7 (35)	1 (100)	–
Family engagement	8 (36.4)	7 (35)	1 (100)	–
Informing patients about delirium	7 (31.8)	7 (35)	–	–
Multiprofessional daily goals	7 (31.8)	6 (30)	1 (100)	–
Going outside, garden, sunshine	6 (27.3)	6 (30)	–	–
Avoidance of bladder tubes/catheters	5 (22.7)	5 (25)	–	–
Undisturbed sleep	3 (13.6)	3 (15)	–	–
Sitter	3 (13.6)	3 (15)	–	–
Provision of ear plugs, sleep glasses	2 (9.1)	2 (10)	–	–
Ground-leveled beds	1 (4.5)	1 (5)	–	–
Activities in patient groups, *e.g*. singing	1 (4.5)	1 (5)	–	–
Animal-assisted therapy	1 (4.5)	1 (5)	–	–

**Table 5S:** Pharmacological management

		South America (n=22)	Argentina (n=20)	Chile (n=1)	Peru (n=1)
Drugs*, n (%)					
	Dexmedetomidine	18 (81.8)	17 (85)	1 (100)	–
	Quetiapine	15 (68.2)	13 (65)	1 (100)	1 (100)
	Haloperidol	14 (63.6)	13 (65)	1 (100)	–
	Lorazepam	12 (54.5)	11 (55)	1 (100)	–
	Risperidone	8 (36.4)	7 (35)	1 (100)	–
	Midazolam	8 (36.4)	8 (40)	–	–
	Reducing of deliriogenic drugs	7 (31.8)	7 (35)	–	–
	Diazepam	5 (22.7)	5 (25)	–	–
	Evaluation by a specialist	3 (13.6)	3 (15)	–	–
	Clonidine	3 (13.6)	3 (15)	–	–
	Phenobarbital	2 (9.1)	2 (10)	–	–
	Melatonin	2 (9.1)	2 (10)	–	–
	Beta-Blockers	1 (4.5)	1 (5)	–	–
	Levodopa	1 (4.5)	1 (5)	–	–
Pharmacological management#, n (%)					
	More individualized approach, depending on patients, and side effects	16 (72.7)	15 (75)	1 (100)	–
	Depending on specific delirium symptoms of each patient	14 (63.8)	14 (70)	–	–
	Reported in handovers	12 (54.5)	12 (60)	–	–
	Included recommendations for withdrawal of delirium-related drugs	11 (50)	10 (50)	1 (100)	–
	Based on a SOP/protocol	7 (31.8)	6 (30)	1 (100)	–
	Included pharmacologists	5 (22.7)	4 (20)	1 (100)	–
	Included psychiatrist or delirium specific liaison team	4 (18.2)	4 (20)	–	–
	More general approach, including a few pharmacological agents	4 (18.2)	3 (15)	–	1 (100)
	Discussed with families in most cases	3 (13.6)	2 (10)	1 (100)	–

* Not reported: Melperone, I do not know;

# Not reported: is discussed with patients.

**Table 6S:** Barriers

Characteristics*, n (%)	South America (n=22)	Argentina (n=20)	Chile (n=1)	Peru (n=1)
Lack of time to educate and train staff	13 (59.1)	11 (55)	1 (100)	1 (100)
Communication gaps between different professionals	12 (54.4)	11 (55)	1 (100)	–
Missing knowledge about delirium	11 (50)	11 (55)	–	–
Not enough motivated staff	9 (40.9)	9 (45)	–	–
Shortage of personnel/staff	9 (40.9)	8 (40)	–	1 (100)
Wrong attitude, delirium not considered important	8 (36.4)	8 (40)	–	–
Lack of awareness	7 (31.8)	7 (35)	–	–
Lack of nonpharmacological interventions	7 (31.8)	6 (30)	–	1 (100)
High cost/ No resources for departmental promotion	6 (27.3)	5 (25)	–	1 (100)
Lack of leadership support	5 (22.7)	5 (25)	–	–
Interprofessional conflicts	4 (18.2)	4 (20)	–	–
No barriers, delirium was regularly assessed	3 (13.6)	2 (10)	1 (100)	–
Patients, assessment of whom was difficult	2 (9.1)	2 (10)	–	–
Other more challenging problems	1 (4.5)	1 (5)	–	–
Lack of pharmacological interventions	1 (4.5)	1 (5)	–	–

* Not reported: No appropriate scores for delirium assessment.

Latin American and Caribbean countries have made strides in research ethics governance; however, they still require support for enforcing a more comprehensive approach. The Pan American Health Organization (PAHO) strategies emphasize resource allocation and capacity building for ethics committees.^(5)^ The WDAD study highlighted the low participation of South American countries in delirium research, prompting questions about the means by which delirium awareness can be enhanced in the region. Implementing additional protocols, increasing knowledge on the disorder, and providing better tools for practice are urgently needed.^(2)^

A recent systematic review showed that only in 19.4% (14/72) of studies, nonpharmacological delirium prevention bundles were actively implemented, with most efforts being part of ICU sedation and analgesia protocols.^(6)^ The reported implementation of protocols (59.1%) and use of validated tools (87.5%) may have been overestimated, indicating the need for increasing delirium awareness.^(2)^

Given the preliminary findings on global delirium assessments, future efforts should prioritize raising awareness and implementing standardized protocols. Increasing delirium awareness is essential for enhancing clinical outcomes worldwide. The education and training of healthcare professionals, alongside the promotion of effective assessment tools, can improve the mechanism by which healthcare systems address delirium. Additionally, adapting interventions to regional contexts, such as effectuating culturally sensitive approaches in South America, can further boost the identification, prevention, and management of delirium, ultimately leading to improved patient outcomes.

Supplementary material with further data on WDAD in South America, including acknowledgment of participants are provided below.

## SUPPLEMENTARY MATERIAL Enhancing delirium awareness in South America: current insights and future perspectives for research and practice

Ricardo Kenji Nawa, Thiago Junqueira Avelino-Silva, Roberta Esteves Vieira de Castro, María Adela Goldberg, Luis Daniel Umezawa Makikado, Fernando Tirapegui Sanhueza, Gabriel Heras-La-Calle, Heidi L. Lindroth, Keibun Liu, Rebecca von Haken, Peter Nydahl

**DOI:** 10.31744/einstein_journal/2024CE1332
